# Values of serum intestinal fatty acid-binding protein, fecal calprotectin, and fecal human β-defensin 2 for predicting necrotizing enterocolitis

**DOI:** 10.1186/s12887-024-04667-5

**Published:** 2024-03-16

**Authors:** Sujia Liu, Yongle Liu, Shuhua Lai, Yingling Xie, Wenlong Xiu, Changyi Yang

**Affiliations:** 1 Department of Neonatology, Fujian Maternity and Child Health Hospital, Fuzhou, People’s Republic of China; 2 Neonatal Intensive Care Unit, Fujian Provincial Hospital, Fuzhou, People’s Republic of China

**Keywords:** β-defensin 2, Calprotectin, I-FABP, NEC, Biomarker

## Abstract

**Background:**

This study aimed to assess the diagnostic potential of serum intestinal fatty acid-binding protein (I-FABP), fecal calprotectin (FC), and fecal human β-defensin 2 (hBD2) in predicting necrotizing enterocolitis (NEC) in preterm infants.

**Methods:**

A prospective cohort of neonates with a gestational age < 32 weeks, suspected of NEC, was enrolled between June 2021 and December 2022. Serum I-FABP, FC, and fecal hBD2 levels were measured upon NEC suspicion, and diagnosis was confirmed through radiological examination or surgical intervention. Diagnostic precision of serum I-FABP, FC, and fecal hBD2 was assessed using a logistic regression model with multiple variables.

**Results:**

The study included 70 neonates (45 males, 25 females), with 30 developing NEC (40% Stage III, *n* = 12; 60% Stage II, *n* = 18) and 40 in the control group. NEC patients exhibited significantly higher serum I-FABP and FC levels (4.76 ng/mL and 521.56 µg/g feces, respectively) than those with other diagnoses (1.38 ng/mL and 213.34 µg/g feces, respectively; p ˂ 0.05 for both biomarkers). Stage II NEC neonates showed elevated fecal hBD2 levels (376.44 ng/g feces) than Stage III NEC neonates and controls (336.87 ng/g and 339.86 ng/g feces, respectively; p ˂ 0.05). No such increase was observed in infants progressing to Stage III NEC. Using a serum I-FABP threshold of > 2.54 ng/mL yielded 76.7% sensitivity, 87.5% specificity, 82.1% positive predictive value (PPV), and 83.3% negative predictive value (NPV). For FC (cutoff > 428.99 µg/g feces), corresponding values were 76.7% sensitivity, 67.5% specificity, 63.9% PPV, and 79.4% NPV.

**Conclusion:**

Serum I-FABP and FC levels are valuable for early NEC detection and provide insights into disease severity. Low fecal hBD2 levels suggest an inadequate response to luminal bacteria, potentially rendering these infants more susceptible to NEC development or exacerbation.

## Introduction

Despite the abundance of available data, the incidence of necrotizing enterocolitis (NEC) diagnosis, primarily affecting infants born prematurely, has not significantly decreased over the past decade [1]. Early identification of intestinal necrosis requiring surgical intervention remains a critical challenge, as the underlying mechanisms of NEC are not fully understood [2]. Major factors influencing the risk of NEC include the immaturity of underdeveloped gut defenses and the immune system [3], complications associated with enteral feeding [4], and the potential for irregular microbial colonization of the gut [5]. Furthermore, the diagnostic process is complicated by the limited accuracy of the current laboratory tests and imaging modalities [6]. In recent years, abdominal sonography has been applied for the early identification of NEC [7]. However, it is not available universally; its manifestations of NEC, such as bowel wall thickening, peritoneal effusion, abnormal bowel movements, portal veins, and emphysema, are not specific to NEC; and ultrasound diagnosis is subjective [8]. Thus, specific biomarkers that can facilitate early NEC diagnosis in high-risk preterm infants are urgently needed, and ideally, these biological indicators should target early stages of NEC pathogenesis. Previous studies have highlighted the advantages of combining multiple biomarkers for NEC diagnosis, focusing on overactive inflammatory reaction [9] and disruption of the gut mucosa, which are characterizations of NEC pathogenesis. Our research targets to enhance the utility of serum intestinal fatty acid-binding protein (I-FABP) as an indicator of compromised gut wall integrity, fecal calprotectin (FC) as an indicator of gut wall inflammation, and fecal human β-defensin (hBD)-2 as an indicator of immune system immaturity.

I-FABP, a biomarker indicating intestinal barrier dysfunction, is a valuable plasma marker for enterocyte damage. This water-soluble cytoplasmic protein (14–15 kDa) exists exclusively in mature bowel enterocytes [10] and is transported into the bloodstream when the integrity of the cellular membrane is compromised. Elevated serum I-FABP levels provide real-time data on the degree of injury to intestinal epithelial cells.

Intestinal inflammation results in the recruitment of neutrophils into the bowel wall, leading to the release of calprotectin, a heterodimeric peptide with a molecular weight of 36 kDa, comprising approximately 60% of the cytosolic content of neutrophils [11]. During intestinal inflammation, calprotectin is readily identified in both fecal and plasma samples [12] and displays significant resilience to degradation by colonic bacteria, underscoring its utility as an indicator for bowel wall inflammation, as seen in cases of inflammatory bowel problems [13].

hBD2 contributes to safeguarding the gut barrier against infections [14]. Notably, hBD2 is quantifiable in fecal samples of both full-term and preterm neonates and its production is unaffected by factors such as gestational age, despite the recognized delays in intestinal colonization among preterm infants, or the feeding method [15].

The principal objective of this study is to assess the diagnostic utility of serum I-FABP, FC, and fecal hBD2 as diagnostic tools for recognizing and predicting the severity of NEC in preterm infants, as well as determining suitable threshold values for conclusive NEC diagnosis.

## Materials and methods

### Patients

**Fig. 1:**
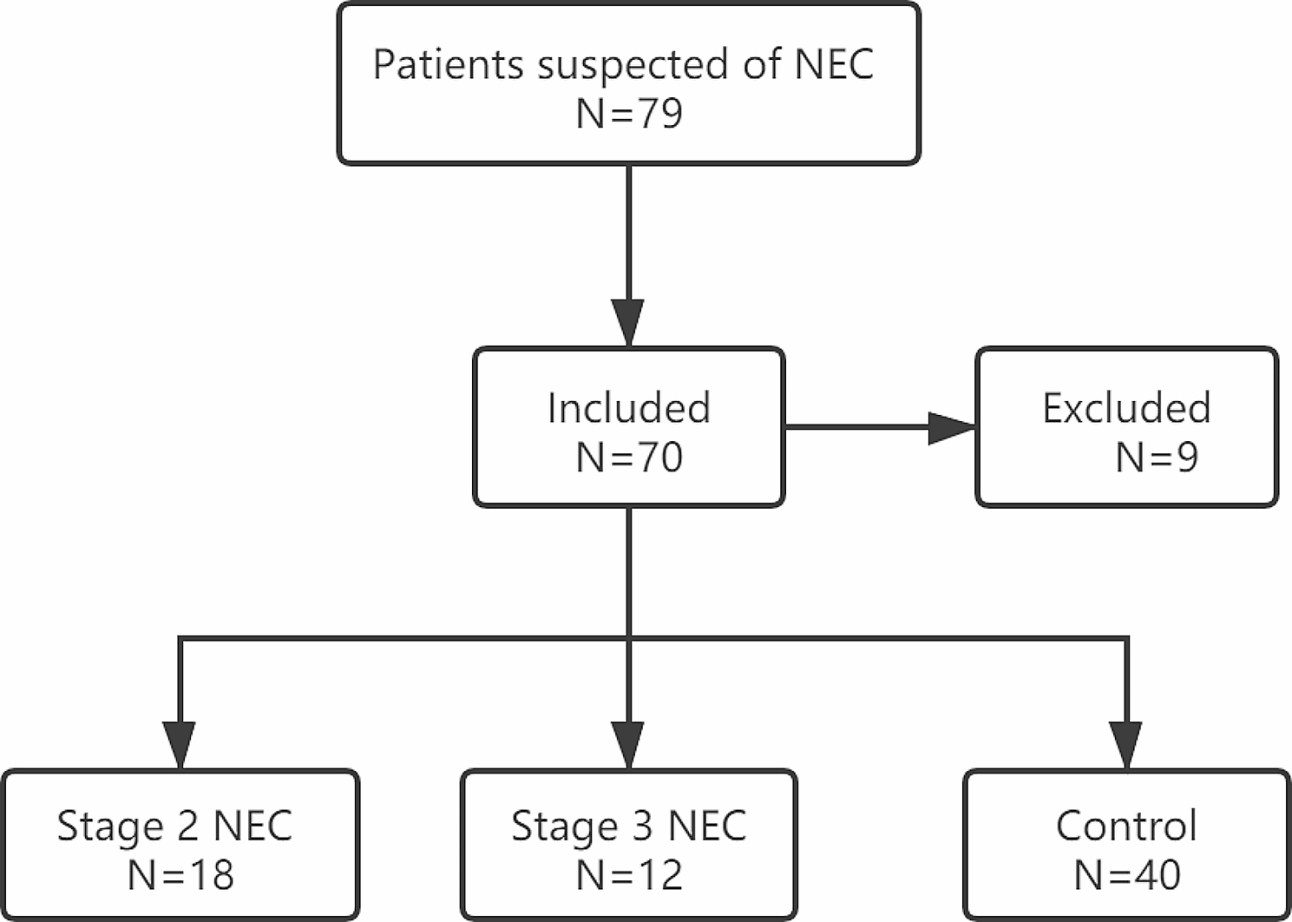
Flow chart of patient inclusion

From January 2021 to June 2022, neonatal intensive care units at Fujian Maternity and Child Health Hospital admitted infants born with a gestational age under 32 weeks. Inclusion criteria comprised neonates with complete clinical data; those suspected NEC with gastrointestinal symptoms, specifically elevated gastric residuals, abdominal swelling, and occult and/or gross bloody stool; those who had successfully retained samples for monitoring plasma I-FABP, FC, and fecal hBD2 levels; and informed consent obtained from guardians. Exclusion criteria included incomplete data, congenital anomalies, metabolic disorders, and lack of parental consent. Accordingly, nine individuals were excluded as they did not provide informed consent. A visual representation of the patient inclusion process is depicted in Fig. 1. Written informed consent was obtained from both parents, and the study protocol received approval from the ethical review board at Fujian Maternity and Child Health Hospital (2,019,070), adhering to the updated Declaration of Helsinki (October 2008, Seoul). The study was carried out in strict compliance with good clinical practice (GCP) principles.

### NEC diagnosis

Criteria for NEC identification considered clinical manifestations and detection of pneumatosis intestinalis or/and portal venous gas on abdominal X-ray imaging, which align with NEC Bell’s Stage II or III classification [16]. Initial diagnosis was established collaboratively by the attending neonatologist and radiologist and subsequently validated by the researchers. Infants diagnosed with NEC at Stage II had at least one imaging finding of pneumatosis intestinalis and portal vein gas, whereas those with disease progression to Stage III presented with ascites and/or pneumoperitoneum. The onset of NEC evaluation was marked by the first abdominal X-ray conducted after the initial clinical suspicion of NEC.

### Laboratory workup

To assess early NEC detection, serum I-FABP, FC, and fecal hBD2 levels were compared with conventional inflammation markers (C-reactive protein, white blood cell count, platelet count) in a blood sample taken when NEC was suspected.

In addition to the standard diagnostic blood analysis conducted using Cell Dyn 1800 (Abbott Diagnostics, Germany), additional peripheral venous blood (1 mL) and fecal matter (100 mg) were collected for research purposes. The blood sample was separated by centrifugation at approximately 2,000 × g for a duration of 10 min to fractionate the plasma, which was subsequently aspirated and transferred into a 0.5-mL Sarstedt tube and stored at − 80 °C for later I-FABP analysis. Fecal samples were frozen at − 80 °C for subsequent FC and hBD2 analysis.

Plasma I-FABP, FC, and fecal hBD2 levels were quantified by a blinded laboratory technician using a commercial colorimetric assay kit (HK406-02) from Hycult Biotech (Uden, The Netherlands). FC and fecal hBD2 levels were determined using ELISA kits sourced from Wuhan Cloud-Clone Corp (China), bearing catalog numbers SEK504Hu No: L201105614 and SEA072Hu No: L200901810, respectively. Sample processing adhered to manufacturer instructions, and biomarker concentrations were measured using certified ELISA kits.

### Statistical analysis

Statistical analysis used SPSS Statistics software version 21.0 (IBM Corp, Armonk, NY, USA). Demographic features were presented as mean ± standard deviation (SD) or median range. In instances of continuous variables conforming to a Gaussian distribution, t-tests were conducted. The Mann-Whitney U-test was conducted for comparing between two quantitative variables, while the Kruskal-Wallis test was deployed for comparing over two quantitative variables for nonparametric data. Categorical variables were evaluated using Chi-square or Fisher’s precise test. Receiver operating characteristic (ROC) plots were generated, with area under the ROC curve (AUC), sensitivity, specificity, and threshold values assessed. Significance was established at *p* < 0.05.

## Results

### Initial attributes of the enrolled newborns

The research cohort comprised 70 neonates (45 males, 25 females). Among the 30 neonates diagnosed with NEC (20 males, 10 females), 18 were categorized as Stage II NEC, and 12 as Stage III NEC. The median sampling day was 16 days, with surgical interventions performed on 19 infants (7 with Stage II NEC, 12 with Stage III NEC). The average bowel resection length in surgical cases was 17.5 cm (range: 10–30 cm), involving the small intestine in eight cases (66.7%), both small and large intestines in two (16.7%), and portions of the colon in two (16.7%). Neonates with NEC had an average gestational age of 29.18 ± 1.63 weeks and a birth weight of 1,275.50 ± 274.47 g. The control group comprised 40 neonates (25 males, 15 females) matched with the study participants in gestational age, postnatal age, and sex. Diagnoses in the control group related to gastrointestinal symptoms included late-onset sepsis (three cases), severe pneumonia (four cases), milk protein allergy (five cases), heart failure (two cases), gastroesophageal reflux (five cases), and preterm infants’ feeding intolerance in the remaining cases, attributed to the immaturity of preterm infants’ intestinal development. The control cohort had an average gestational age of 29.51 ± 1.46 weeks and a birth weight of 1,356.00 ± 270.28 g, with no substantial disparities in gestational age or birth weight between the two groups. A summary of the demographic and clinical attributes is provided in Table 1.

**Table 1:** Patient demographics

	NEC	Control	Χ^2^/t/U	*p* value
Number of infants, n	30	40		
Bowel resected, n	12			
Length of bowel resected, M (IQR), cm	17.5 (10–30)			
Bowel resection region				
Small bowel, n (%)	8 (66.7%)			
Larger bowel, n (%)	2 (16.7%)			
Small bowel + larger bowel, n (%)	2 (16.7%)			
NEC Stage II, n (%)	18 (60%)	-		
NEC Stage III, n (%)	12 (40%)	-		
Sex, male, n (%)	20 (66.6%)	25 (62.5%)	0.130	0.719
Gestational age, x ± SD, w	29.18 ± 1.63	29.51 ± 1.46	0.893	0.375
Birth weight, x ± SD, g	1,275.50 ± 274.47	1,356.00 ± 270.28	−1.225	0.225
Twin, n (%)	8 (26.7%)	9 (22.5%)	0.162	0.687
Birth asphyxia, n (%)	7 (23.3%)	7 (17.5%)	0.365	0.546
Premature rupture of membranes, n (%)	11 (36.7%)	15 (37.5%)	0.005	0.947
Days of sampling, M (IQR), d	16.0 (12.3–22.0)	16.0 (14.0–21.0)	636.000	0.669

d: day; g: gram; IQR: interquartile range; NEC: necrotizing enterocolitis; SD: standard deviation; w: week

### Routine laboratory investigations of study groups

Table 2 presents the comparison of laboratory metrics. No significant differences were observed in the median WBC count (5.41 [0.89–32.94] vs. 8.03 [3.22–20.71] ×10^9^/L; *p* = 0.055) or platelet (PLT) count (260 [98–632] vs. 274.5 [79–561] ×10^9^/L; *p* = 0.25] between -the NEC and control groups. The NEC group exhibited significantly elevated C-reactive protein (CRP) levels compared with the control group (1.66 [0.46–93.40] mg/L vs. 0.53 [0.30–31.53] mg/L; *p* < 0.05; Table 2). However, no statistically significant difference in CRP levels was observed between the NEC Stage II and control groups. Notably, a significant difference was observed between the NEC Stage III and control groups (Table 3).

**Table 2:** Laboratory parameters of neonates according to study groups

	NEC (*n* = 30)	Control (*n* = 40)	U statistic	*p* value
WBC (×10^9^/L), M (IQR)	5.41 (0.89–32.94)	8.03 (3.22–20.71)	438.000	0.055
PLT (×10^9^/L), M (IQR)	260 (98–632)	274.5 (79–561)	503.000	0.250
CRP (mg/L), M (IQR)	1.66 (0.46–93.40)	0.53 (0.30–31.53)	412.500	0.026
FC (ug/g), M (IQR)	521.56 (113.55–4358.23)	213.34 (32.15–1036.29)	210.000	0.000
hBD2 (ng/g), M (IQR)	357.56 (309.86–391.46)	339.86 (319.57–368.45)	449.000	0.073
I-FABP (ng/mL), M (IQR)	4.76 (1.245–13.238)	1.38 (0.083–4.961)	124.000	0.000

CRP: C-reactive protein; FC: fecal calprotectin; hBD2: human β-defensin 2; I-FABP: intestinal fatty acid-binding protein; IQR: interquartile range; PLT: platelet; WBC: white blood cell

**Table 3:** Comparison of plasma I-FABP, FC, hBD2, and CRP levels among NEC neonates according to modified bell’s system stages and the control set

	CRP (mg/L)	FC (ug/g)	hBD2 (ng/g)	I-FABP (ng/mL)
Stage II NEC (*n* = 18)	0.57 (0.50–14.87)	482.99 (113.55–1552.18)	376.44 (352.27–391.46)	3.36 (1.245–6.745)
Stage III NEC (*n* = 12)	14.24 (0.46–93.4)	818.06 (328.40–4358.23)	336.87 (316.33–372.67)	9.28 (5.085–13.238)
Control (*n* = 40)	0.53 (0.30–31.53)	213.34 (32.15–1036.29)	339.86 (319.57–368.45)	1.38 (0.083–4.961)
*p* value	0.016^**a**^	0.000 ^**a**^	0.000 ^**a**^	0.000 ^**a**^
	0.204^**b**^	0.101 ^**b**^	0.000 ^**b**^	0.017 ^**b**^
	1.0^**c**^	0.014 ^**c**^	0.000 ^**c**^	0.002 ^**c**^
	0.012^**d**^	0.000 ^**d**^	0.474 ^**d**^	0.000 ^**d**^

^a^ Kruskal–Wallis test for comparison among three groups.

^b^ Stage II NEC vs. stage 3 NEC for dual comparison by Mann–Whitney U-test.

^c^ Stage II NEC vs. control for dual comparison by Mann–Whitney U-test.

^d^ Stage III NEC vs. control for dual comparison by Mann–Whitney U-test.

CRP: C-reactive protein; FC: fecal calprotectin; hBD2: human β-defensin 2; I-FABP: intestinal fatty acid-binding protein; NEC: necrotizing enterocolitis.

### Plasma I-FABP, FC, and hBD2 levels among the studied sets

The median concentrations of serum I-FABP and FC were significantly higher in infants with suspected NEC (4.76 µg/mL and 521.56 µg/g, respectively) than in control subjects (1.38 ng/mL and 213.34 µg/g, respectively), despite similar postnatal ages (*p* < 0.05 for both). Conversely, fecal hBD2 showed no statistically significant variation between the control and NEC groups (357.56 ng/g and 339.86 ng/g, respectively; Table 2). Plasma I-FABP and FC levels in neonates diagnosed with NEC were significantly different from those in the control group. Stage III NEC neonates exhibited the highest median levels (*p* < 0.05 for both) of I-FABP (3.36 ng/mL, 9.28 ng/mL, and 1.38 ng/mL for Stage II NEC, Stage III NEC, and controls, respectively) and FC (482.99 ng/g, 818.06 ng/g, and 213.34 ng/g for Stage II NEC, Stage III NEC, and controls, respectively). However, no statistically significant distinction in FC levels was observed between Stage II NEC and Stage III NEC neonates (Table 3).

**Fig. 2:**
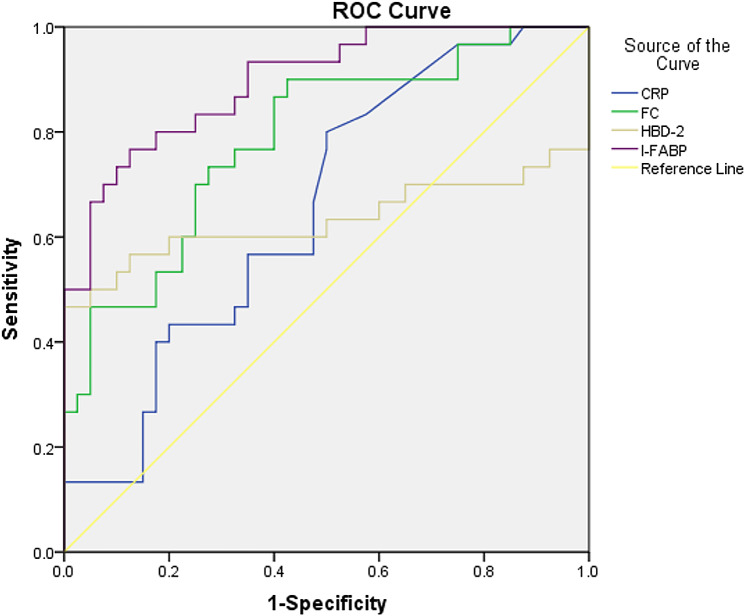
ROC Curve Comparing the Accuracy of CRP, FC, hBD2, and I-FABP Levels in Discriminating NEC Neonates from Controls. CRP: C-reactive protein; FC: fecal calprotectin; hBD2: human β-defensin 2; I-FABP: intestinal fatty acid-binding protein; ROC: receiver operating characteristic

To assess the diagnostic precision of CRP, FC, fecal hBD2, and serum I-FABP levels, ROC curves were generated. Serum I-FABP outperformed other markers in distinguishing NEC patients from controls, boasting an AUC of 0.897 (95% confidence interval [CI] 0.825–0.968). The AUC for FC was 0.787 (95% CI 0.679–0.895), while it was 0.656 (95% CI 0.529–0.784) for CRP and 0.633 (95% CI 0.474–0.79) for fecal hBD2. Specific threshold values were selected to maximize sensitivity and specificity for each marker. For serum I-FABP, a cutoff of 2.54 ng/mL yielded 76.7% sensitivity and 87.5% specificity. In the case of FC, a cutoff of 428.99 µg/g creatinine resulted in 76.7% sensitivity and 67.5% specificity. For CRP, a threshold of 0.575 mg/L provided 63.3% sensitivity and 52.5% specificity (Fig. 2; Table 4).

**Table 4:** Accuracy of CRP, FC, hBD2, and I-FABP prediction of NEC.

	Cutoff value	Sensitivity (%)	Specificity (%)	PPV (%)	NPV (%)	AUC (95% CI)
CRP	0.575 (mg/dL)	63.3	52.5	50	65.7	0.656 (0.529–0.784)
FC	428.99 (ug/g)	76.7	67.5	63.9	79.4	0.787 (0.679–0.895)
hBD2	339.60 (ng/g)	63.3	50.0	48.7	35.5	0.633 (0.474–0.790)
I-FABP	2.54 (ng/mL)	76.7	87.5	82.1	83.3	0.897 (0.825–0.968)

AUC: area under the receiver operating characteristic curve; CI: confidence interval; CRP: C-reactive protein; FC: fecal calprotectin; hBD2: human β-defensin 2; I-FABP: intestinal fatty acid-binding protein; NEC: necrotizing enterocolitis; NPV: negative predictive value; PPV: positive predictive value

## Discussion

NEC diagnosis relies on the identification of pneumatosis intestinalis, sometimes accompanied by additional clinical signs such as absent bowel sounds and escalating metabolic acidosis. Advanced stages of NEC involve multiple organ complications and escalating gastrointestinal distress, marked by pronounced abdominal distension or palpation-induced pain. The widely employed Bell’s staging system categorizes NEC into suspected (Stage I), defined (Stage II), and advanced (Stage III) [17]. While advanced NEC stages often display more conspicuous clinical symptoms, early stages can manifest with nonspecific indications. Consequently, identifying a biomarker capable of predicting or diagnosing early-stage NEC is crucial for timely intervention before progression. Among gastrointestinal fatty acid-binding proteins, serum I-FABP has emerged as a potential diagnostic indicator for NEC [18]. Liebermann et al. pioneered the quantitative radioimmunoassay for human I-FABP, suggesting its potential as a diagnostic indicator for early intestinal mucosal vulnerability, as seen in NEC [19]. Subsequent investigations have continued to underscore I-FABP’s promise as an early diagnostic tool for NEC. Our research underscores that plasma I-FABP measurements at symptom onset can discern patients with initially vague symptoms who ultimately develop definitive NEC. We established a serum I-FABP threshold at 2.54 ng/mL, emphasizing high specificity to mitigate the risk of misdiagnosis. A recent meta-analysis encompassing 572 infants (262 with NEC and 310 healthy) revealed a positive correlation between serum or urine I-FABP levels and NEC presence, displaying a standardized mean difference of 2.88 and 95% CI of 2.09–3.67 (*p* < 0.01) [20]. Increased blood levels of I-FABP reflects enterocyte damage. Results from two large, academic pediatric surgical centers that plasma I-FABP levels at disease onset are strongly associated with the length of intestinal resection in surgical NEC. The median length of bowel resection was 10 (range: 2.5–50) cm and 17 (range: 0–51) cm, respectively, in the two centers, and median I-FABP levels were 53 (range: 6.3–370) ng/mL and 4.2 (range: 1.1–15.4) ng/mL in plasma [21]. Our findings substantiate these conclusions and offer additional insights into the association between I-FABP levels and infants’ Bell’s stages, as well as align with previous findings of I-FABP as a specific marker for the early identification of severe NEC (Bell’s Stage III) [22, 23]. The distinction between NEC and non-NEC patients chiefly stemmed from cases of NEC that ultimately progressed to complicated NEC. In our study, individuals initially suspected of NEC but ultimately not diagnosed served as controls, mirroring real-world clinical scenarios. Our results affirm the value of I-FABP as a diagnostic and prognostic tool, even at the emergence of nonspecific gastrointestinal symptoms. When comparing I-FABP with conventional inflammation markers and other potential indicators (such as FC and hBD2), our research unveiled significantly elevated I-FABP levels in NEC-infected infants versus controls.

Calprotectin, predominantly found in neutrophils, is released into the intestinal lumen during accelerated leukocyte turnover [24]. Its presence in feces signifies neutrophilic inflammation within the bowel, especially in neonates at risk of NEC [25]. A prior investigation indicated swift calprotectin assessments in preterm infants, highlighting its potential for early NEC detection [26]. Among preterm neonates, FC levels demonstrated a transient upsurge concurrent with intestinal symptoms, which declined upon treatment or ailment resolution [27, 28]. There is no uniform conclusion that the FC level for the diagnosis of NEC. For preterm infants with gastrointestinal symptoms, suggested fecal FC thresholds were 363 µg/g for mild enteropathy and 636 µg/g for severe enteropathy [29]. Swift and dependable NEC diagnosis remains paramount for prompt intervention. While clinicians traditionally depend on clinical manifestations for NEC identification, novel biomarkers and imaging approaches substantiate the diagnosis [30]. In a preliminary inquiry by Carroll et al., seven preterm neonates with confirmed NEC reported significantly elevated FC levels compared with healthy infants matched for gestational age [31]. Bin-Num et al. found that FC levels were elevated with NEC (3,000 µg/g stool [2075–7875]) vs. without (195 µg/g stool [110–440]; *p* < 0.001), and ROC curves indicated that 480 µg/g as the threshold for NEC diagnosis had 100% sensitivity and 84.6% specificity. They concluded that monitoring FC in children with suspected NEC is beneficial to its early diagnosis [26]. Thuijls et al. further reported the effectiveness of FC measures in diagnosing NEC, pinpointing an optimal threshold of 286.2 µg/g for distinguishing NEC-afflicted neonates from those with suspected NEC and alternative conclusive diagnoses, yielding 93% specificity and 86% sensitivity [32]. Our research reported markedly higher FC levels in neonates with NEC than in controls. However, no noteworthy disparity in FC was observed between NEC Stage II and III neonates. Calprotectin exhibited moderate predictive utility for NEC diagnosis. A threshold of 428.99 µg/g was reported to exhibit 76.7% sensitivity and 67.5% specificity in NEC diagnosis. Furthermore, the applicability of FC assessment in aiding NEC diagnosis might be confined to scenarios where stool specimens are unattainable.

The intestinal epithelium responds to microbial stimuli by actively producing defensins and cathelicidins [33]. HBDs constitute a cluster of evolutionarily conserved antimicrobial peptides crucial for innate host defense across various mucosal surfaces, including the gastrointestinal tract. Although epithelial cells inherently express hBD1, the induction of hBD2, hBD3, and hBD4 depends on diverse inflammatory and bacterial stimulants [34]. HBD2, a key protein in the innate immune response, protects humans against invading pathogens of bacterial, viral, fungal, and parasitical origin. Its pivotal role in enhancing immunity has been demonstrated in infants [35]. The compromised response of premature neonates to potential infections reflects their unique profiles of underdeveloped innate and adaptive immune reactions, rendering them vulnerable to substantial infections and contributing to the pathogenesis of ailments specific to this demographic, such as NEC [36]. One prevalent hypothesis concerning the pathophysiology of NEC revolves around the underdeveloped innate mucosal defense of the intestine [37]. Inadequate fecal hBD2 expression emerges as a substantial factor in NEC development. Remarkably, fecal hBD2 levels can be readily measured and consistently detected in the feces of both full-term and preterm neonates. Moreover, fecal hBD2 production seems impervious to gestational age, despite the recognized delay in intestinal colonization in preterm infants, as well as the feeding mode. Campigotto et al.‘s study reported elevated fecal hBD2 levels in Bell’s Stage III NEC neonates [15], aligning with the findings of Liu et al.’s study [38]. Additionally, Jenke et al. demonstrated significantly elevated fecal hBD2 levels in infants with moderate NEC preceding clinical symptoms [39], different from those in infants who continued to develop severe NEC. Elevated fecal hBD2 levels, indicative of robust intestinal immune responses, correlated with moderate disease progression. Conversely, in severe NEC cases, low hBD2 expression was associated with diminished Toll-like receptor 4 (TLR4)/MD2 expression, implying an inadequate response to luminal bacteria, potentially fostering NEC susceptibility [39]. Our study revealed that fecal hBD2 levels exhibited no statistically discernible distinctions between Stage III NEC neonates and controls. However, hBD2 levels were notably higher in Stage II NEC neonates than in controls and Stage III NEC neonates. While hBD2 may possess restricted value for the early prediction of NEC, lower hBD2 levels may be indicative of an insufficient response to luminal bacteria, predisposing infants to NEC. Alternatively, this observation could be attributed to the effective sample size in our study, necessitating further evidence to confirm this marker’s superiority.

Our study has a few limitations. First, it is important to emphasize the importance of continuous biomarker monitoring as NEC progresses. Additionally, the study was conducted at a single medical center, with a relatively limited number of participants, particularly among infants with severe NEC. Evaluating the early predictive and prognostic value of each index, particularly in critically ill and surgical infants with Stage III NEC, remains challenging. Therefore, multicenter clinical trials with larger cohorts are essential to validate the predictive significance of the identified biomarkers within the scope of this study.

## Conclusion

Serum I-FABP and FC emerge as valuable screening tools for NEC in clinically suspected cases, offering insights into disease severity. Furthermore, lower hBD2 levels suggest an inadequate response to luminal bacteria, potentially increasing the susceptibility of infants to NEC development or aggravation. Our research indicated that serum I-FABP exhibited the highest predictive value for early NEC, with both high sensitivity and specificity, while FC demonstrated a moderate predictive value. Thus, fecal hBD2, while predicting the lowest exacerbation, provides some reflection of the intestinal immune status.

## Data Availability

Availability of data and materialsThe novel findings and contributions of this research are documented in the manuscript and appendix data. For any additional questions, please contact the corresponding author.
